# Derivatives of Cinnamic Acid Esters and Terpenic Diversity in Volatiles of Thirty-Six Sand Ginger (*Kaempferia galanga* L.) Accessions of Eastern India Revealing Quality Chemovars

**DOI:** 10.3390/molecules27031116

**Published:** 2022-02-08

**Authors:** Subhashree Singh, Suprava Sahoo, Bhaskar Chandra Sahoo, Manaswini Dash, Sanghamitra Nayak, Basudeba Kar

**Affiliations:** Centre for Biotechnology, School of Pharmaceutical Sciences, Siksha ‘O’ Anusandhan Deemed to Be University, Kalinga Nagar, Ghatikia, Bhubaneswar 751003, Odisha, India; subhashreesingh1992@gmail.com (S.S.); supi.sos2000@gmail.com (S.S.); bhaskar.sahoo877@gmail.com (B.C.S.); dashmanaswini97@gmail.com (M.D.); sanghamitran24@gmail.com (S.N.)

**Keywords:** *Kaempferia galanga*, essential oil, GC-MS, cinnamic acid esters, AHC, PCA

## Abstract

The essential oil of *Kaempferia galanga* L. commonly known as sand ginger has increased its demand in national and international market for decades. Cinnamic acid esters like ethyl-*p*-methoxy cinnamate (EPMC) and ethyl cinnamate (EC) are major constituents in its essential oil. In spite of the high demand for the plant as raw material, identification of quality chemovars having high essential oil (EO) yield and constituents is still at an infant stage. With this in mind, we have evaluated the EO yield of 36 accessions from three provinces of Eastern India, which varied within a range of 0.41 ± 0.01 to 2.63 ± 0.03 *v*/*w*. Further, a total of 65 compounds were detected by gas chromatography and mass spectrometry (GC-MS) with area percentages varying from 76.16 to 97.3%. EPMC was found to be the major component in 14 accessions with area percentages varying from 10.7% to 41.1%, whereas other 22 accessions showed EC as the major constituent, varying from 16% to 29.1%. Further, a diversity study among accessions was performed by agglomerative hierarchical clustering (AHC) and principal component analysis (PCA) analysis based on the abundance of identified constituents, which categorized all 36 accessions into three clusters. Thus, the present study helps to identify quality chemovar K.g16 and K.g14 with respect to oil yield and constituents, respectively, which could be used to guide commercial cultivation and further improvement of the taxa.

## 1. Introduction

Plant secondary metabolites play a significant role in the development of important drugs and cosmetics as they possess distinct physiological actions. With time, their uses have proven safety, efficacy, cultural acceptability and lesser side effects [1]. *Kaempferia galanga* L. (sand ginger) of the Zingiberaceae family is one such plant which has seen increased demand in the medicinal industry for decades due to the presence of bioactive secondary metabolites. Globally, *K. galanga* have been extensively cultivated in India, Java, Malaysia, Africa, China, and Sri Lanka [2]. In India, states such as West Bengal, Karnataka, Tamilnadu, and Kerala majorly cultivate this plant as Kapoor kachri [3,4]. According to the earlier reports, this species has biological and pharmacological activities which includes nematicidal, larvicidal, antineoplastic, and vasorelaxant effects [5,6,7,8,9,10]. Traditionally, the rhizomes and the root tubers are used for treating wounds, piles, coughs, fever, skin diseases, epilepsy, bronchitis, rheumatism, asthma, malaria, and splenic disorders. For these reasons, *K. galanga* rhizomes have been known as stimulant, expectorant, diuretic, carminative, antihypertensive, larvicidal, and repellent in traditional remedies [11,12].

Phytochemical studies of *K. galanga* essential oil have revealed the presence of multiple compounds like ethyl-*p*-methoxy cinnamate (EPMC), ethyl cinnamate (EC), *δ*-3-carene, camphene, 1,8-cineole, pentadecane, borneol, etc. [13]. EPMC and EC were reported to be important bioactive constituents as these are highly cytotoxic to HeLA cells [14]. Xue and Chen [15] have also reported the anticancer properties of *K. galanga*. It has also been reported that *K. galanga* plant containing of EPMC inhibits resistant strains of *Mycobacterium tuberculosis* [16]. Such an immense curative ability of this plant enhances its significance in the medicinal industries. Due to high demand, the cost of essential oil on the global market ranges from US$ 400–700/kg [17]. 

Plant essential oils are complex mixture of different volatile constituents like terpenoids, phenyl polypropanoids, alkane hydrocarbons, alcohols, aldehydes, ketones, etc. which can be analyzed by gas chromatography mass spectrometry (GC-MS). This has been successfully used for characterizing chemical constituents of other different important plant essential oils [18,19,20,21,22]. Diverse GC-MS analyses of *K. galanga* rhizome essential oil have been reported earlier from different Asian countries on the basis of different chemovars, but in all the reports, cinnamic acid ester derivatives were identified as major constituents in rhizome essential oil that is either EC or EPMC [2,7,13,23,24,25,26].

*K. galanga* is one of the important medicinal plants which were included in the list of 112 medicinal herbs and spices, released by the International Standardization Organization (ISO). A lack of scientific attention and awareness have made this plant a comparatively unrecognized one [17,27], but with increasing demand for this plant as a raw material, growers are gradually getting focused on cultivating quality material to enhance their livelihood. The focus on *K. galanga* is aimed at selecting accessions with high oil yield and quality constituents so as to provide superior raw materials [28].

Quality chemovars possess not only high oil yields but are also rich in bioactive constituents which could be used to develop value added products. Selection of such chemovars can be achieved by diversity study, as the oil yield and quality vary from place to place. Thus, diversity studies are an efficient approach towards selection of quality chemovars that can be utilized for future crop improvement. This plant can be cultivated under optimum conditions with a view to essential oil extraction for commercial purposes. The optimum environmental conditions for cultivation of *K. galanga* are well drained, fertile and rich moist soil. Although this wild plant grows well at elevations up to 1000 m in partial shade areas like open forest or forest edges with humid climate, it can also be cultivated at other regions with optimum agronomic conditions for industrial applications [29]. Identification of high yielding essential oil chemovars with quality constituents are required to meet the commercial requirements. 

With all these concerns in mind, the current study was designed to select high oil yielding chemovars of *K. galanga* from three provinces (Odisha, West Bengal, Assam) of Eastern India. A diversity study with respect to EO composition of *K. galanga* by GC-MS analysis was also performed to explore new characteristics among the various accessions. 

## 2. Materials and Methods

### 2.1. Plant Material

**Figure 1:**
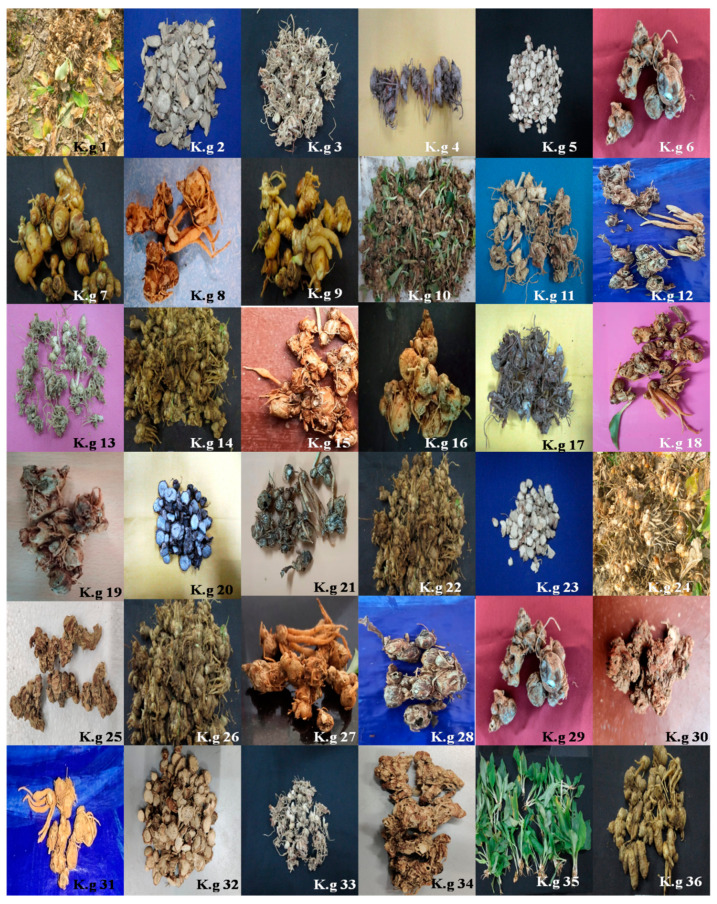
*K. galanga* collected from different locations of three provinces of Eastern India viz. Odisha, West-Bengal (W.B) and Assam. Odisha accessions—K.g1, K.g2, K.g3, K.g4, K.g14, K.g15, K.g16, K.g17, K.g18, K.g21, K.g22, K.g26, K.g27, K.g28; W.B accessions—K.g5, K.g6, K.g7, K.g8, K.g9, K.g11, K.g12, K.g13, K.g23, K.g24, K.g25, K.g29, K.g30; Assam accessions—K.g10, K.g19, K.g20, K.g31, K.g32, K.g33, K.g34, K.g35.

**Figure 2:**
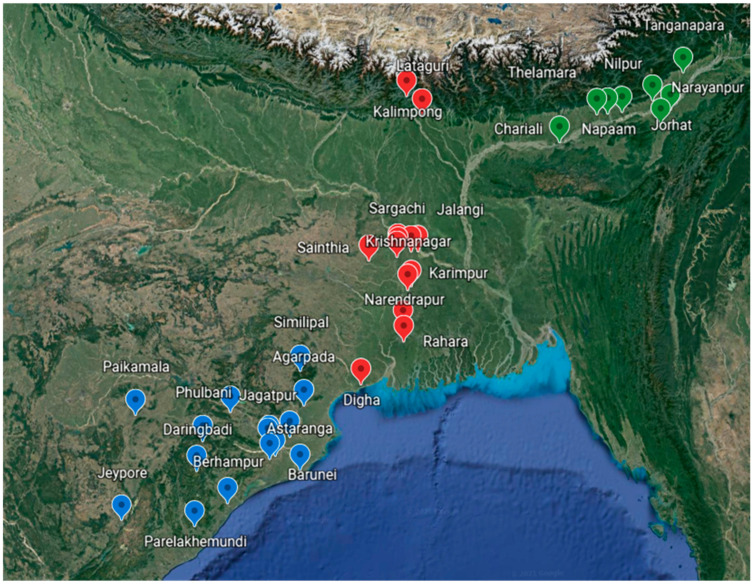
Map showing different regions of sample collection from Eastern India (Blue tags—Odisha provinces, red tags—West-Bengal provinces, green tags—Assam provinces).

Thirty-six accessions of *K. galanga* were collected during the months of October to December in 2017 from three different provinces of Eastern India (Odisha, West Bengal, and Assam) (Figure 1). The collected accessions were identified and authenticated by a renowned taxonomist at Regional Plant Resource Center (RPRC), Bhubaneswar and Centre for Biotechnology (CBT), Siksha ‘O’ Anusandhan Deemed to be University, Bhubaneswar (Table 1, Figure 2). Herbarium samples of accession K.g1–20 were submitted to the Plant Taxonomy and Conservation Department, RPRC, Bhubaneswar and those of K.g21–36 were submitted to CBT, Siksha ‘O’ Anusandhan Deemed to be University, Bhubaneswar. 

### 2.2. Essential Oil Extraction

The rhizomes of *K. galanga* were shade dried and 100 g from each accession were taken for essential oil (EO) isolation by a hydro-distillation method. The process was conducted using a Clevenger-type apparatus for a period of 3–4 h. Further, the EOs were treated with anhydrous sodium sulfate to remove any water content. The purified EOs were then stored in amber glass vials at 4 °C until further analysis. EO isolations from each accession were performed in triplicate to confirm the yield reproducibility. EO yield was calculated on dry weight basis and statistical analysis has been carried out to understand the significance of variation. Analysis of variance (ANOVA) and Tukey’s honestly significant difference (HSD) test at *p* < 0.05 were carried using the Minitab 17 statistical software (Minitab Inc., State College, PA, USA).

### 2.3. GC-MS Analysis

*K. galanga* essential oils of different accessions were analyzed using GC-MS. Thiswas performed using a Clarus 580 gas chromatograph (Perkin Elmer Inc., Waltham, MA, USA) equipped with a SQ8S mass spectrometry detector. Helium was used as the mobile phase at a flow rate of 1ml/min. Neat oil (0.1 µL) was injected into the system for performing the analyses. The column used was an Elite-5 column (30 m length with 0.25 mm internal diameter and 0.25 µm of film thickness). Initially, the oven temperature was set at 60 °C for 1 min and then heated at 3 °C/min to 220 °C with a 6 min hold period. The total runtime was 60 min. The transfer interface and source temperatures were set at 250 °C and 150 °C, respectively. Scanning was performed over a mass scan range of 50–600 amu with EI mode at an ionization voltage of 70 eV. Turbo Mass TM software 6.1.0 (PerkinElmer Inc.) was used for acquiring the ion chromatogram and mass spectra. Retention Index (RI) was calculated by determining RT (Retention Time) of straight chain *n*-alkanes (Sigma Aldrich, St. Louis, MO, USA) with similar operating conditions. The mass spectra of detecting compounds were compared with the inbuilt NIST/EPA/NIH mass spectra library (NIST 11). The identified compounds were further validated by comparing RI values obtained from the experiment with data reported in the literature and Adams library [30]. EPMC (Cayman Chemicals, Ann Arbor, MI, USA) and EC (Sigma Aldrich) standards were injected in GC-MS to authenticate the identification of both compounds in all accessions. Both the standards (0.1 µL) were injected into the system while keeping all parameters the same as for the analytes.

### 2.4. Statistical Analysis

To correlate the similarity and relationship between different accessions of *K. galanga*, agglomerative hierarchical clustering (AHC) and principal component analysis (PCA) were accomplished using Minitab 17 statistical software. Constituents having area percentages greater than 1% were considered for both the analysis. For AHC, Pearson distance was calculated in accordance with the Ward linkage variance minimizing method towards analyzing linear correlation among samples for hierarchical clustering [31]. To accomplish the PCA, the correlation matrix was resolved, which transformed 15 variables into 15 principal components (PCs) [32]. Eigen values of the principal components are the constants that increase or decrease the eigenvectors (variance) along its span when transformed linearly. The variables that are being used in PCs are taken as loadings and the individual transformed observations are as scores. 

## 3. Result and Discussion

### 3.1. Essential Oil Extraction

The essential oils from 36 accessions of *K. galanga* rhizomes were isolated and the mean oil yield was evaluated by using one way ANOVA followed by Tukey’s HSD test at a confidence interval of 95%. The oil yield varied within a range of 0.41 ± 0.01%–2.63 ± 0.03% on a dry weight basis (Table 2) The highest oil yield was found to be in the K.g16 (2.63 ± 0.03% *v*/*w*) accession, whereas the lowest oil yield (0.41 ± 0.01% *v*/*w*) was obtained for the K.g15 accession. The K.g16 chemovar with the highest essential oil yield was collected from Daringbadi (Kandhamala District, Odisha) at 914 m altitude, where the annual average rainfall is 1176.1 mm and temperature varies from 4–32°C.

The oil yield of *K. galanga* in this current study was higher than in previous reports about India [13,25,33]. Raina et al., reported oil yields ranging from 0.3 to 1.9% and 0.83 to 2.19% for samples collected from different districts of Southern Western Ghats in Karnataka and Kerala states, respectively [13]. A new accession from the North East region having a yield of 2.38% on average has also been reported. Similarly, in other Asian countries variations in oil yield have also been reported, with highest yield reported so far being 2% [25,33]. The grouping of the accessions denotes that there were significant variations in oil yield among different accessions on the basis of different geographical locations (Table 2). There are several factors which are responsible for the variation in essential oil production like seasonal variation, mechanical or chemical injuries, geographical variation and environmental factors [34]. The accessions taken in this study were all collected at the beginning of the winter season that is from October–December 2017, hence seasonal variation has less chance of causing EO yield variations. Other factors mentioned above could be collectively responsible for the same. A higher range of altitudes plays an important role as well in varied EO yield, as reported for many species like *Hedychium coronarium*, *Zingiber zerumbet*, *Curcuma longa*, *Zingiber officinale*, *Myristica fragans,* etc. [34,35,36,37]. K.g16 having the highest essential oil yield with quality constituents could be used for large scale cultivation and subsequently for commercial production of essential oil. 

### 3.2. GC-MS Analysis

**Figure 3:**
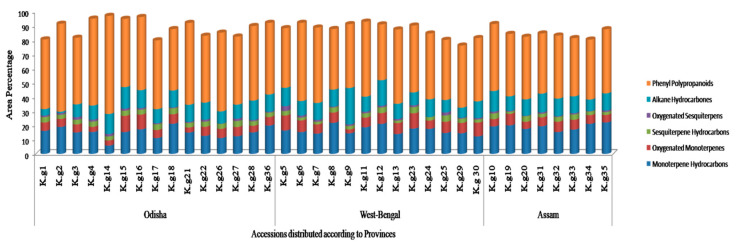
Classification of different group of compounds based on their abundance (area %) present in essential oil of *K. galanga* rhizomes collected from three different provinces.

The phyto-constituents of *K. galanga* EOs were analyzed by GC-MS and a total of 65 compounds were detected. The area percentages of all identified constituents in different accessions fall within the range of 76.16–98.9%. In all accessions, phenyl polypropanoids (EPMC and EC) constituted the major components, varying from 39.26 to 69%. In Odisha accessions, the abundance of phenyl polypropanoids was high (43.17–69%) followed by monoterpene hydrocarbons (5.8–21.21%), alkane hydrocarbons (1.2–15.19%), oxygenated monoterpenes (3.53–11.38%), sesquiterpenes hydrocarbon (2.8–4.2%) and oxygenated sesquiterpenes (0.17–1.84%) (Table S1a from Supplementary Materials). Similarly in West-Bengal samples phenyl polypropanoids represent the major area percentages (39.26–55%), followed by alkane hydrocarbons (7.14–25.64%), monoterpene hydrocarbons (12.43–21.82%), oxygenated monoterpenes (2.73–10.75%), sesquiterpene hydrocarbons (1.72–4.44%) and oxygenated sesquiterpenes (0.09–3.44%) (Table S1b from Supplementary Materials). Likewise, in Assam accessions the abundance of phenyl polypropanoids was also high (40.97–47.11%), followed by monoterpene hydrocarbons (15.32–22.14%), alkane hydrocarbons (8.14–14.52%) and oxygenated monoterpenes (5.01–8.08%), sesquiterpene hydrocarbons (1.54–4.01%) and oxygenated sesquiterpenes (0.3–1.25%) (Figure 3; Table S1c from Supplementary Materials).

Accessions of Odisha province were rich in EPMC (20.14–41.13%) as major constituent in almost all accessions, except K.g16 and K.g18 where EC (14.9–29.9%) was found to be the major one. The other top constituents were pentadecane (1–14.7%) *δ*-carene (1.31–12.28%), 1,8-cineole (1.51–7.13%) etc. (Table 3a and Table S1a from Supplementary Materials). Among West Bengal provinces, all accessions except K.g5 contained EC (20.9–29.14%) as the major constituent, followed by EPMC (10.7–27%), pentadecane (7.14–24.93%), *δ*-carene (5.87–12.28%) 1,8-cineole (2.09–6.54%), etc. (Table 3b and Table S1b from Supplementary Materials). All the accessions of the Assam region have EC (21.54–26%) as the major constituent, followed by EPMC (17.16–22.21%), pentadecane (8.14–14.52%), *δ*-carene (7.1–11.76%), 1,8-cineole (3.21–5.21%), etc. (Table 3c and Table S1c from Supplementary Materials). Standards of EPMC and EC were injected for authentic identification of these two major bioactive constituents in all samples. The GC chromatograms of two standards gave peaks at retention times (RT) of 37.32 and RT 26.35, respectively (Figure S1 from Supplementary Materials). Structures of all identified constituents have been indicated in the Supplementary Materials (Table S2). In this study, the chemical compositions identified in rhizome EO of *K. galanga* accessions closely resembled earlier reports on same plant from Malaysia [33], Sri Lanka [38], and India [23]. All accessions of *K. galanga* have cinnamic acid esters as major constituents and were more or less similar to each other with respect to other chemical constituents. However, significant variations were found with respect to the area percentages of the constituents. A similar pattern of findings has also been reported in other studies where the major constituent was either EC or EPMC or their *trans* isomers like ethyl *trans*-cinnamate, ethyl *trans*-*p*-methoxy cinnamate and methyl cinnamate [2,10,13,26]. 

**Figure 4:**
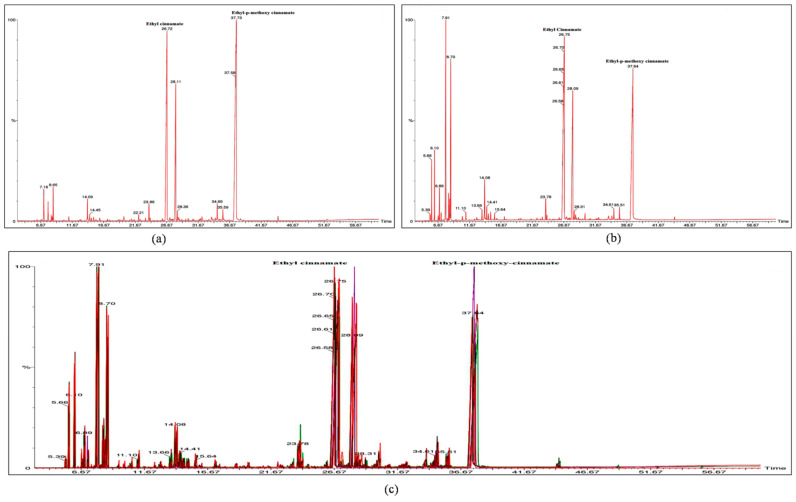
GC-MS chromatogram of *K*. *galanga* rhizome essential oil with different constituents. (**a**) Chromatogram of K.g14 showing EPMC as major constituent and EC as second major constituent (**b**) Chromatogram of K.g16 showing EC as major constituent and EPMC as second major constituent (**c**) Consensus GC-MS fingerprint of *K. galanga* accessions collected from three different provinces showing similar pattern.

Higher abundance of EPMC is one of the important criteria towards selection of quality chemovars as it is a highly active constituent with cytotoxic, proapoptotic, anti-inflammatory, analgesic, anti-neoplastic, and anti-oxidant like activities [24,39]. Similarly the abundance of bioactive constituent EC has almost equal importance in the selection of quality chemovars. In this study, GC-MS analysis revealed two important chemovars, K.g16-EPMC and K.g14-EC (Figure 4a,b). However, other constituents were also identified and mentioned in Table S1. The GC-MS peaks of all accessions were merged to develop a consensus fingerprint towards confirming the authenticity of accessions collected from different provinces (Figure 4c). The consensus GC-MS chromatogram could be used to check for adulteration in *K. galanga* rhizome essential oil. 

### 3.3. Statistical Analysis

**Figure 5:**
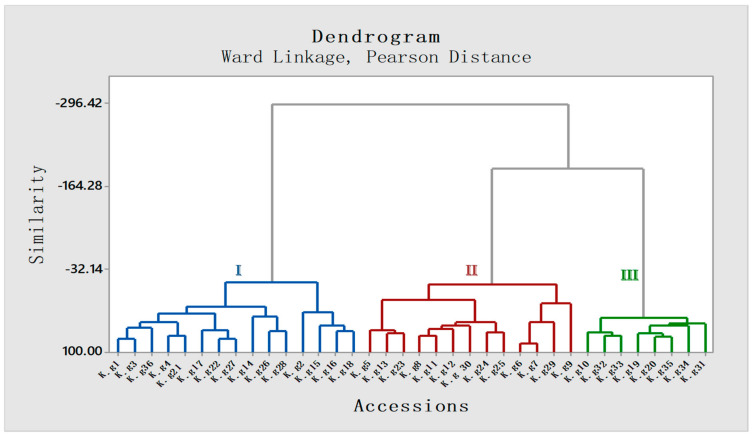
Agglomerative hierarchical clustering (AHC) based dendrogram of 36 accessions of *K. galanga* based on Ward Linkage (Pearson distance). All 36 accessions have been distributed into 3 clusters on the basis of similarity in area percentage of constituents among different accessions. Cluster I: Odisha, Cluster II: West-Bengal, Cluster III: Assam (III).

To establish a correlation among different accessions with respect to quality constituents, agglomerative hierarchical clustering (AHC) and principal component analysis (PCA) were performed using the Minitab statistical software. According to the AHC analysis, the dendrogram group I represents the Odisha province accessions, whereas groups II and III represent the West-Bengal and Assam province accessions, respectively (Figure 5).

**Figure 6:**
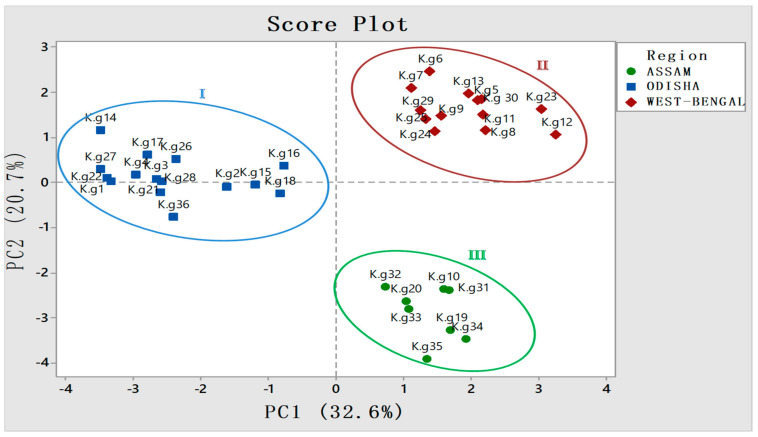
Three different provinces and their respective accessions falling into different quadrants in score plot of principal component analysis (PCA) showing significant diversity w.r.t constituents having area % ≥ 1.

To further validate the result, PCA analysis was employed, which categorized all 36 accessions into three different groups according to different province (Figure 6). The regions of West-Bengal and Assam fell sharply in two different quadrants, whereas accessions from Odisha share another quadrant with slight deviations of a few constituents. This may be due to the collection of samples from diverse eco-regions of Odisha (Figure 6).

**Figure 7:**
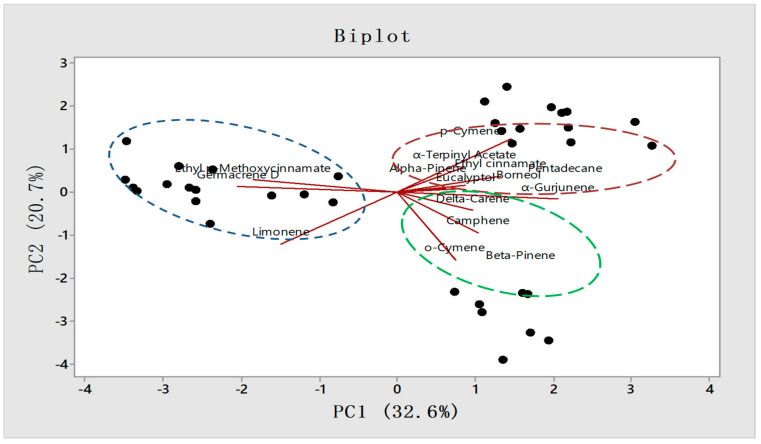
PCA Biplot graph representing individual constituents responsible for the variation among provinces.

The PCA analysis biplot presented the constituents responsible for the variations among 36 accessions of three provinces (Figure 7). The key constituents responsible for the variation in Odisha province are EPMC (20.14– 41.1%), germacrene D (1–1.9%), and limonene (1–2.1%). In West Bengal *p*-cymene (1–1.42%), α-terpinyl acetate (0.12–1.08%), were mostly responsible for the variation from the other two provinces. Similarly, in Assam accessions compounds like *o*-cymene (1.01–1.9%), *β*-pinene (1.01–1.31%), and *α*-gurjunene (1–1.56%) have been identified as responsible for the variations among the other two provinces. 

The variations obtained in phyto-constituents are quite natural as these accessions have been taken from different geographical regions. Due to their different geographical regions, the species are exposed to different exogenous factor-related stresses which lead to the production of different secondary metabolites for their defense [40]. Several reports in other species are also available in which variation in constituents was discussed for different eco-regions [35,36]. Identifying a single parameter responsible for such variations is a very cumbersome process as collective exogenous factors such as soil, pH, altitude, growing site precipitation, etc. may be responsible for such variations as discussed by other researchers [22,29]. All these parameters may not be present at a single region, which leads to variation in essential oil yield and its constituents. PCA and AHC were used for the first time as pattern recognizing methods to study the chemical diversity analysis of *K. galanga* essential oil. This approach it has also been successfully employed to analyze the correlation and variation in different crops [35,36,41]. 

## 4. Conclusions

It can be concluded from the current study that there are variations in the yield and chemical composition of different *K. galanga* accessions collected from Odisha, West-Bengal, and Assam provinces of India. The impact of environmental factors of different geographical regions may be the possible reason to account for the observed variations in essential oil yield and quality constituents. All these accessions were characterized by a maximum amount of phenyl polypropanoids, which were cinnamic acid esters. Two chemovars obtained in this study (K.g16 and K.g14) with highest essential oil yield and quality constituent could be utilized for large scale cultivation with a view towards industrial utilization.

## Figures and Tables

**Table 1:** Geographical characteristics of *K. galanga* accessions collected from Eastern India.

Accessions	Locality	Province	Voucher Specimen	Longitude (E)	Lattitude (N)	Altitude (m)	T_max_ (°C)	T_min_ (°C)	A.A.P * (mm)
K.g1	Barunei	Odisha	11,053	85°38′40′′	20°10′39′′	109	34.5	21.9	1500
K.g2	Jagatpur	Odisha	11,054	85°36′02′′	20°27′52′′	40	32.6	21.9	1515
K.g3	Patrapada	Odisha	11,055	85°45′37′′	20°14′34′′	47	31.7	21.9	1505
K.g4	Berhampur	Odisha	11,056	84°47′00′′	19°18′15′′	25	29.7	22	1194
K.g5	Jalangi	West Bengal	11,057	88°41′26′′	24°7′41′′	473	29.6	18.6	1459
K.g6	Domkol	West Bengal	11,058	88°32′49′′	24°7′31′′	23	25.4	22.8	1530
K.g7	Krishnanagar	West Bengal	11,062	88°28′55′′	23°24′04′′	19	30.9	18.9	1353
K.g8	Karimpur	West Bengal	11,059	88°33′24′′	23°28′15′′	10	30	18.6	1434
K.g9	Narendrapur	West Bengal	11,063	88°23′57′′	22°26′25′′	9	30.3	19.6	1645
K.g10	Narayanpur	Assam	11,064	93°49′58′′	26°56′31′′	83	32	20.8	1518
K.g11	Berhampore	West Bengal	11,065	85°14′59′′	20°05′40′′	23	30.7	18.7	1347
K.g12	Kalimpong	West Bengal	11,066	88°27′21′′	27°04′13′′	1067	22.1	12	2395
K.g13	Sargachi	West Bengal	11,067	88°14′58′′	24°01′17′′	24	30.7	18.8	1365
K.g14	Jeypore	Odisha	11,068	82°34′07′′	18°53′00′′	592	30.3	19	1587
K.g15	Astaranga	Odisha	11,069	86°16′12′′	19°58′48′′	8	29.7	22.1	1337
K.g16	Daringbadi	Odisha	11,070	84°7′60′′	19°53′60′′	914	32	4	1176.1
K.g17	Phulbani	Odisha	11,071	84°14′47′′	20°28′31′′	26	31.6	19	1323
K.g18	Parelakhemundi	Odisha	11,072	84°05′55′′	18°50′4′′	145	46	10	1925.6
K.g19	Chariali	Assam	11,073	91°44′45′′	26°10′20′′	51	39.5	14.1	2054
K.g20	Jorhat	Assam	11,075	94°13′12′′	26°45′0′′	116	28.9	16.6	2324
K.g21	Similipal	Odisha	1018/CBT	86°15′26′′	21°51′41′′	622	32.4	20.2	1596
K.g22	Paikamal	Odisha	1019/CBT	82°48′45′′	20°55′18′	273	35.2	20.4	1435
K.g23	Lataguri	West Bengal	1020/CBT	88°46′49′′	26°43′10′′	123	27.9	16.9	3242
K.g24	Lalbagh	West Bengal	1021/CBT	88°16′10′′	24°09′39′′	24	29.8	18.4	1343
K.g25	Rahara	West Bengal	1022/CBT	88°23′13′′	22°43′33′′	12	30.5	19.5	1569
K.g26	Basantapur	Odisha	1023/CBT	84°48′52′′	21°03′04′′	215	30.6	18.7	1540
K.g27	Agarpada	Odisha	1024/CBT	86°20′30′′	21°12′36′′	42	31.9	20.6	1530
K.g28	Chhatia	Odisha	1025/CBT	86°03′21′′	20°36′34′′	39	31.9	21.1	1519
K.g29	Sainthia	West Bengal	1026/CBT	87°40′03′′	23°56′59′′	50	31	18.7	1328
K.g30	Digha	West Bengal	1027/CBT	87°31′04′′	21°37′235′′	11	30.8	20.4	1519
K.g31	Napaam	Assam	1028/CBT	92°49′31′′	26°42′14′′	73	28.8	17.5	1836
K.g32	Thelamara	Assam	1029/CBT	92°35′09′′	26°41′40′′	77.1	28.9	17.3	1903
K.g33	Tanganapara	Assam	1030/CBT	94°33′26′′	27°28′21′′	106	29	17.2	2111
K.g34	Nilpur	Assam	1031/CBT	93°08′30′′	26°43′37′′	83.8	29	17.2	2326
K.g35	Chinatoli	Assam	1032/CBT	93°59′18′′	26°29′06′′	102.4	29	16.6	2782
K.g36	Athagarh	Odisha	1033/CBT	85°37′51′′	20°31′06′′	63	32.2	21.4	1484

Note: * AAP—Average annual precipitation.

**Table 2:** Essential oil yield percentage of *K.* *galanga* from different accessions.

Accessions	Province	Locality	Oil Yield (%) (Mean ± SD) ^a^
K.g1	Odisha	Barunei	0.65 ± 0.03
K.g2	Odisha	Jagatpur	1.5 ± 0.03
K.g3	Odisha	Patrapada	0.63 ± 0.04
K.g4	Odisha	Berhampur	0.84 ± 0.04
K.g5	West Bengal	Jalangi	2.41 ±0.01
K.g6	West Bengal	Domkol	1.84 ± 0.05
K.g7	West Bengal	Krishnanagar	1.63 ± 0.03
K.g8	West Bengal	Karimpur	2.41 ± 0.04
K.g9	West Bengal	Narendrapur	2.35 ± 0.02
K.g10	Assam	Lakhimpur	0.92 ± 0.02
K.g11	West Bengal	Berhampore	0.51 ± 0.01
K.g12	West Bengal	Kalimpong	1.92 ± 0.16
K.g13	West Bengal	Sargachi	0.84 ± 0.05
K.g14	Odisha	Jeypore	2.3 ± 0.01
K.g15	Odisha	Astaranga	0.41 ± 0.01
Kg16	Odisha	Daringbadi	2.63 ± 0.03
K.g17	Odisha	Phulbani	1.33 ± 0.03
K.g18	Odisha	Parelakhemundi	0.76 ± 0.03
K.g19	Assam	Chariali	1.22 ± 0.02
K.g20	Assam	Jorhat	1.83 ± 0.03
K.g21	Odisha	Similipal	2.03 ± 0.06
K.g22	Odisha	Paikamal	1.63 ± 0.03
K.g23	West Bengal	Lataguri	1.23 ± 0.04
K.g24	West Bengal	Lalbagh	1.43 ± 0.02
K.g25	West Bengal	Rahara	1.22 ± 0.03
K.g26	Odisha	Basantapur	0.82 ± 0.06
K.g27	Odisha	Agarpada	1.24 ± 0.03
K.g28	Odisha	Chhatia	1.77 ± 0.1
K.g29	West Bengal	Sainthia	1.88 ± 0.01
K.g30	West Bengal	Digha	1.77 ± 0.02
K.g31	Assam	Napaam	2.01 ± 0.02
K.g32	Assam	Thelamara	1.98 ± 0.03
K.g33	Assam	Tanganapara	1.32 ± 0.03
K.g34	Assam	Nilpur	1.26 ± 0.03
K.g35	Assam	Chinatoli	1.31 ± 0.04
K.g36	Odisha	Athagarh	1.82 ± 0.05

^a^ Mean ± standard deviation (SD).

**Table 3:** Major constituents (GC area% ≥ 1) of *K. galanga* rhizome essential oil collected from different locations of (**a**) Odisha province, (**b**) West-Bengal province, (**c**) Assam province.

(a)																		
Sl No.	Compound	RI ^a^	RI ^b^	Odisha														
				K.g1	K.g2	K.g3	K.g4	K.g14	K.g15	K.g16	K.g17	K.g18	K.g21	K.g22	K.g26	K.g27	K.g28	K.g36
1	α-Pinene	932	932	1.3	1.91	1.1	1.19	1	1.7	1.53	2.1	2.1	1	1.3	1.25	1.45	1.2	1.31
2	Camphene	949	946	1.49	3.03	1.58	1.86	1	3.07	1.97	1.12	2.59	1.9	1.01	2.04	1.76	1.25	3.15
3	*δ*-Carene	1011	1008	8.59	10.9	8.28	9.64	1.31	7.87	9.97	5.1	12.28	9.37	6.5	5.12	3.17	8.56	10.06
4	Limonene	1028	1024	1.14	1.09	1.09	1.13	1	1.04	1	1	1.14	1.8	1.76	1	1.41	1.64	1.67
5	1,8-Cineole	1032	1026	3.23	3.64	3.08	2.37	1.51	6.03	7.13	3.76	5.35	1.62	4.12	3.17	4.13	2.09	4.12
6	Borneol	1170	1165	1	1.54	1.07	1.23	1.19	2.38	1.9	1.23	1.29	1.11	1	2.31	1.16	2.07	1.19
7	*β*-Elemene	1383	1389	0.33	-	0.68	-	0.16	-	1.03	0.99	-	-	0.89	-	-	-	-
8	Cyperene	1394	1398	-	-	-	-	-	-	-	-	1.1	-	-	-	-	0.67	-
9	*n*-Tetradecane	1396	1400	0.23	-	0.2	-	1.03	-	0.22	-	-	-	0.76	-	-	0.13	0.12
10	*α*-Gurjunene	1402	1409	0.54	0.26	0.2	0.64	0.17	0.9	-	0.88	0.26	0.95	-	1.01	0.87	1	0.67
11	Ethyl Cinnamate	1467	1465	14.9	28	18.87	22.31	27.9	19	29.7	17.12	23.03	27.27	18.98	24.41	19.09	21.02	17.98
12	Germacrene D	1673	1484	1.07	1.06	1.16	1.17	1	1.11	1.21	1	1.31	1.16	1.6	1.5	1.87	1	1
13	Pentadecane	1504	1500	4.33	1	8.42	9.94	12.4	14.7	11.9	9.87	11.98	12.1	10.89	8.59	9.87	13.54	12.1
14	8,9 Epoxide Cadalene	1677	1674	0.29	-	0.49	-	1	0.59	-	-	-	-	-	-	-	-	-
15	Ethyl *p*-Methoxycinnamate	1769	1760	31.8	33.9	25.36	38.79	41.13	29	25.35	31.25	20.14	30.26	27.32	30.98	28.76	31.41	32.4
**(b)**																		
**Sl No.**	**Compound**	**RI ^a^**	**RI ^b^**	**West-Bengal**														
				**K.g5**	**K.g6**	**K.g7**	**K.g8**	**K.g9**	**K.g11**	**K.g12**	**K.g13**	**K.g23**	**K.g24**	**K.g25**	**K.g29**		**K.g30**	
1	α-Pinene	932	932	1.57	1	1.01	2.1	1	1.66	1.81	1.77	1.98	1.68	1.89	1.09		1.78	
2	Camphene	949	946	2.67	1.14	1.2	2.59	1.9	2.57	2.63	2.75	3.71	2.4	2.18	1.21		1.32	
3	*β*-Pinene	977	974	0.62	0.34	0.35	1.01	0.3	0.84	1.01	0.72	1.09	0.14	1.2	1		0.89	
4	*δ*-Carene	1011	1008	8.29	10	9.9	12.28	9.37	10.38	11.98	6.91	7.09	10.09	5.87	8.67		6.32	
5	*p*-Cymene	1021	1020	1.42	1.39	1	1.24	1.24	1.38	1	1.14	1.08	1	1	1		1	
6	*o*-Cymene	1023	1022	-	-	-	1.2	-	-	0.84	-	-	-	-	-		-	
7	Limonene	1028	1024	0.96	0.65	0.45	0.14	0.35	0.15	0.2	0.32	0.98	0.87	0.76	0.98		0.21	
8	1,8-Cineole	1032	1026	4.44	5	4	5.35	1.62	4.82	5.42	3.56	6.14	2.09	3.21	5.15		6.54	
9	Borneol	1170	1165	1.8	1.01	1	1.82	1.11	1.81	2	1.54	1.89	1.6	2.19	1.15		2.3	
10	*α*-Terpinyl Acetate	1350	1346	1.01	1	1	-	-	-	-	0.98	1.08	1.01	0.14	0.89		0.12	
11	*α*-Gurjunene	1402	1409	1.19	1	1	1.01	1	1.31	1.16	1.03	1.12	1.04	1	1.03		1.3	
12	Ethyl Cinnamate	1467	1465	20.9	28	27	23.03	27.2	28.2	28.56	28.74	29.14	26.16	24.14	27.67		27	
13	Pentadecane	1504	1500	12.5	10	12	11.98	24.93	10.6	18.01	10.33	9.09	12.47	8.78	7.14		11.78	
14	Germacrone	1673	1693	1	-	0.45	-	1	-	-	-	-	-	-	-		-	
15	Ethyl *p*-Methoxycinnamate	1769	1760	21.1	27	26	19.58	17.66	24.56	10.7	23.54	17.87	20.14	18.14	15.99		17.56	
**(c)**																		
**Sl No.**	**Compound**	**RI ^a^**	**RI ^b^**	**Assam**														
				**K.g10**		**K.g19**		**K.g20**		**K.g31**		**K.g32**		**K.g33**		**K.g34**		**K.g35**
1	α-Pinene	932	932	1.72		1		1		1.2		1.45		2.1		1.98		1.1
2	Camphene	949	946	2.31		4.32		2.99		3.7		2		2.5		4.65		3.1
3	*β*-Pinene	977	974	1.01		1.23		1.14		1.01		1.15		1.31		1.16		1.2
4	*δ*-Carene	1011	1008	11.76		10.5		9.87		11.2		7.1		8.13		9.45		10.13
5	*p*-Cymene	1021	1020	-		-		0.43		-		0.21		0.16		0.31		1.9
6	*o*-Cymene	1023	1022	1.07		1.1		1.01		1.09		1.21		1.16		1.31		1.9
7	Limonene	1028	1024	1.08		1.19		1		1.03		1.16		1.31		1.22		1.7
8	1,8-Cineole	1032	1026	3.51		5.21		3.21		3.76		4.64		4.12		3.99		3.5
9	Borneol	1170	1165	1.6		1.6		1.46		1.24		1.31		1.65		1.76		1.58
10	*α*-Terpinyl Acetate	1350	1346	-		0.4		0.23		1		0.89		0.67		0.22		-
11	*α*-Gurjunene	1402	1409	1.56		1.01		1.23		1		1		1.14		1.09		1.2
12	Ethyl Cinnamate	1467	1465	24.9		25.8		23.89		23.2		24.17		21.54		25.02		26
13	Pentadecane	1504	1500	14.52		10.12		11.54		13.1		12.46		11.24		8.14		12.1
14	Ethyl *p*-Methoxycinnamate	1769	1760	22.21		18.14		20.14		19.2		20.14		19.43		17.16		19.1

^a^ RI, Retention indices on Elite-5 column, experimentally determined using homologous series of C8–C20 *n*-alkanes. ^b^ RI; Retention index taken from literature [30].

## Data Availability

Not applicable.

## Supplementary Materials

The following are available online. Table S1 (a) Chemical profiling of *Kaempferia galanga* rhizome essential oil collected from different provinces of Odisha, (b) Chemical profiling of *Kaempferia galanga* rhizome essential oil collected from different provinces of West-Bengal, (c) Chemical profiling of *Kaempferia galanga* rhizome essential oil collected from different provinces of Assam. Table S2. Chemical Structure of all identified constituents from *K. galanga* rhizome essential oil. Figure S1. Authentic identification of EC and EPMC constituents in analytes by co-injecting with known standards through GC-MS. (a): Chromatogram of EC standard (b): Consensus GC-MS fingerprint of *K. galanga* accessions collected from three different provinces showing similar pattern. (c): Chromatogram of EPMC standard.
