# Supplementary material for: Derivatives of Cinnamic Acid Esters and Terpenic Diversity in Volatiles of Thirty-Six Sand Ginger (Kaempferia galanga L.) Accessions of Eastern India Revealing Quality Chemovars
Source: Molecules. 2022 Feb 8;27(3):1116. doi: 10.3390/molecules27031116 (PMC8840707; doi:10.3390/molecules27031116)
Supplement: Supplementary file 1 [file molecules-27-01116-s001.zip › molecules-1446853-supplementary.pdf]

Supplementary Materials

Derivatives of Cinnamic Acid Esters and Terpenic Diversity in Volatiles of Thirty-Six Sand Ginger (*Kaempferia galanga* L.) Accessions of Eastern India Revealing Quality Chemovars

Table S1. Chemical profiling of *Kaempferia galanga* rhizome essential oil collected from different provinces.

(a)

Sl no.	Compound	RI[a]	RI[b]	Odisha														
				K.g1	K.g2	K.g3	K.g4	K.g14	K.g15	K.g16	K.g17	K.g18	K.g21	K.g22	K.g26	K.g27	K.g28	K.g36
1	Tricyclene	922	921		0.24				0.22		0.12		0.053		0.19	0.65	0.13	
2	α-Thujene	923	924									0.13						
3	α-Pinene	932	932	2	1.91	1.3	1.19	1	1.7	1.53	2.1	2.1	1	1.3	1.25	1.45	1.2	1.31
4	Camphene	949	946	1.6	3.03	1.58	1.86	1	3.07	1.97	1.12	2.59	1.9	1.01	2.04	1.76	1.25	3.15
5	Sabinene	970	969		0.32				0.67	0.24		0.79			0.27	0.68	0.8	
6	β-Pinene	977	974	0.48	0.77	0.5	0.46		0.63	0.93	0.85	0.9	0.31	0.76	0.5	0.95	0.34	0.87
7	Myrecene	987	988	0.22	0.33	0.22	0.24	0.85		0.22	0.26	0.5	0.15	0.64		0.6	0.68	0.98
8	Dehydroxy *Trans* Linalool Oxide	993	991															
9	δ-Carene	1011	1008	8.59	10.9	8.28	9.64	1.31	7.87	9.97	5.1	12.28	9.37	6.5	5.12	3.17	8.56	10.06
10	*p*-Cymene	1021	1020	0.83	0.39		0.72		0.38	0.99	0.34	0.25	0.5		0.23	0.78	0.65	0.67
11	*o*-Cymene	1023	1022			0.77		0.27								0.6		
12	Limonene	1028	1024	2	1.09	2.1	1.13	1	1.04	1	1	1.14	1.8	1.76	1	1.41	1.64	1.67
13	1,8-Cineol	1032	1026	3.3	3.64	3.08	2.37	1.51	6.03	7.13	3.76	5.35	1.62	4.12	3.17	4.13	2.09	4.12
14	γ-Terpinene	1054	1054															
15	Mentha-2,4,8-Diene (*p*)	1098	1085	0.23	0.24	0.31		0.21			0.42	0.48				0.9		0.9
16	Terpinoline	1097	1086				0.2							0.51				0.14
17	*p*-1,3,8-Menthatriene	1097	1108							0.32					0.13			
18	*Cis* Limonene Oxide	1129	1132						0.22					0.6				0.13
19	Borneol	1170	1165	2	1.54	2	1.23	1.19	2.38	1.9	1.23	1.29	1.11	1	2.31	1.16	2.07	1.19
20	*m*-Cymen-8-ol	1176	1176	0.32		0.38		0.3	0.72	0.52			0.4					0.1
21	*p*-Cymen-8-ol	1182	1179						0.38	0.26					0.41			
22	α-Terpineol	1190	1186			0.19		0.18	0.24	0.32	0.17			0.15		0.19		
23	Verbenone	1208	1204		0.4				0.56	0.23	0.31		0.4	0.4		0.44	0.5	0.12

24	4-Methylene-Isophorone	1215	1216						0.21									
25	*Endo*-Fenchyl Acetate	1213	1218					0.17						0.14	0.08	0.57		0.22
26	Car-3-en-2-one	1242	1244						0.37									
27	Sabinyl Acetate (*trans*)	1288	1289															
28	*p*-Vinyl Guaiacol	1302	1309					0.26	0.27									
29	Myrtenyl Acetate	1323	1324															
30	α-Terpinyl Acetate	1350	1346	0.28							0.14				0.44			0.13
31	α-Longipinene	1357	1350	0.244				0.37	0.22									
32	β-Elemene	1383	1389	0.33		0.68		0.16		1.03	0.99			0.96				
33	Cyperene	1394	1398									1.1					0.67	
34	*n*-Tetradecane	1396	1400	0.233		0.2		1.03		0.22				0.86			0.13	0.12
35	α-Gurjunene	1402	1409	0.54	0.26	0.2	0.64	0.17	0.9		0.88	0.26	0.95		1.01	0.93	1	0.67
36	β-Cedrene	1403	1419	0.22		0.2	0.16						0.16				0.13	0.15
37	α-Guaiene	1431	1437								0.13				0.11			
38	α-Humulene	1451	1452				0.41											
39	Ethyl Cinnamate	1467	1465	16	27.98	18.87	22.31	29.9	19	26.09	17.12	23.03	27.27	19.7	24.41	19.09	21.02	17.98
40	*Cis*-Muurola-4(14),5-Diene	1475	1465						0.43									
41	ϒ-Muurolene	1476	1478			0.2		0.26		0.19								
42	Germacrene D	1673	1484	1.4	1.06	1.16	1.17	1	1.11	1.21	1	1.31	1.16	1.7	1.5	1.87	1	1
43	δ-Selinene	1494	1492															
44	Pentadecane	1504	1500	4.33	1	8.42	9.94	12.4	14.7	11.9	9.87	11.98	12.1	10.89	8.59	9.87	13.54	12.1
45	Epizonarene	1508	1501	0.35	0.34	0.37		0.5		0.44	0.41			0.22				
46	γ-Cadinene	1513	1513	0.28	0.23	0.27	0.54	0.21	0.69	0.3		0.63	0.57	0.18	0.31	0.48	0.12	0.079
47	Cubebol	1523	1514								0.59			0.27				
48	β-Curcumene	1517	1514						0.38									
49	δ-Cadinene	1529	1522		0.41		0.42					0.53	0.36			0.15		0.89
50	α-Cadinene	1526	1537									0.36						0.17
51	α-Copaene-11-ol	1540	1539															
52	Selina3,7(11)-Diene	1555	1545	0.41	0.66	0.21	0.34	0.16	0.34	0.3	0.62		0.01	0.53	0.61	0.77	0.23	0.08
53	Guaiol	1606	1594															
54	Carotol	1606	1594	0.5		0.4		0.22			0.12		0.41			0.87	0.31	0.41
55	*Epi*-α-Muurolol	1646	1640					0.37		0.3								
56	α-Muurolol	1651	1644	0.4	0.98	0.5	0.31		0.41			0.59		0.79				
57	Pogostol	1662	1651			0.45		0.15		0.17								
58	Citronellyl Tiglate	1669	1666							0.51								
59	8,9 Epoxide Cadalene	1677	1674	0.2		0.49		1	0.59									

60	Germacrone	1673	1693											0.31	0.17	0.8	0.61	0.17
61	*n*-Heptadecane	1694	1700			0.36		0.6	0.49	0.51	0.1			0.31				
62	Zerumbone	1720	1732				0.9											
63	Ethyl-*p*-Methoxy Cinnamate	1769	1760	33	33.87	28	38.79	41.13	29	25.35	31.25	20.14	30.26	27.32	30.98	28.76	31.41	32.4
64	*n*-Pentadecanol	1773	1773		0.2		0.16			0.11			0.31					
65	Pimaradiene	1950	1948		0.23		0.27						0.12		0.33		0.28	
	Total Identified (%)			80.29	92.02	81.69	95.4	98.88	95.22	96.16	80	87.73	92.29	82.93	85.16	83.03	90.36	91.98
	Monoterpene Hydrocarbons			16.1	19	15.06	15.44	5.8	15.36	17.29	11.2	21.213	15.03	12.67	11.19	12.43	15.12	19.94
	Oxygenated Monoterpenes			5.89	5.6	5.65	3.6	3.6	11.38	10.36	5.61	6.64	3.53	6.41	6.41	6.49	4.66	6.01
	Sesquiterpene Hydrocarbons			3.7	3.1	3.29	3.68	2.8	3.64	3.47	4.03	4.19	3.21	3.59	3.54	4.2	3.15	3.039
	Oxygenated Sesquiterpens			1.1	1.2	1.84	1.37	1.7	1.43	1.09	0.71	0.59	0.72	1.37	0.17	1.67	0.92	0.58
	Alkane Hydrocarbones			4.5	1.2	8.98	9.94	14	15.19	12.6	9.97	11.98	12.1	12.06	8.59	9.87	13.67	12.22
	Polypropanoids			49	61.85	46.87	61.1	71	48	51.4	48.4	43.17	57.53	47.02	55.39	47.85	52.43	50.38

(b)

Sl no.	Compound	RI[a]	RI[b]	West-Bengal												
				**K.g5**	**K.g6**	**K.g7**	**K.g8**	**K.g9**	**K.g11**	**K.g12**	**K.g13**	**K.g23**	**K.g24**	**K.g25**	**K.g29**	**K.g 30**
1	Tricyclene	922	921	0.19				0.05	0.18	0.18	0.21	0.15	0.31	0.89	0.11	0.14
2	α-Thujene	923	924						0.14	0.17						
3	α-Pinene	932	932	1.57	1	1.01	2.1	1	1.66	1.81	1.77	1.98	1.68	1.89	1.09	1.78
4	Camphene	949	946	2.67	1.14	1.2	2.59	1.9	2.57	2.63	2.75	3.71	2.4	2.18	1.21	1.32
5	Sabinene	970	969	0.51			0.28		0.2	0.23						
6	β-Pinene	977	974	0.62	0.34	0.35	1.01	0.3	0.84	1.01	0.72	1.09	0.14	1.2	1	0.89
7	Myrecene	987	988	0.2			0.5	0.15	0.39	0.47	0.21	0.56	0.13	0.8	0.28	0.67
8	Dehydroxy *Trans* Linalool Oxide	993	991						0.09	0.11						
9	δ-Carene	1011	1008	8.29	10	9.9	12.28	9.37	10.38	11.98	6.91	7.09	10.09	5.87	8.67	6.32
10	*p*-Cymene	1021	1020	1.42	1.39	1	1.24	1.24	1.38	1	1.14	1.08	1	1	1	1
11	*o*-Cymene	1023	1022				1.2			0.84						
12	Limonene	1028	1024	0.96	0.65	0.45	0.14	0.35	0.15	0.2	0.32	0.98	0.87	0.9	0.98	0.21
13	1,8-Cineol	1032	1026	4.44	5	4	5.35	1.62	4.82	5.42	3.56	6.14	2.09	3.21	5.15	6.54
14	γ-Terpinene	1054	1054		0.79				0.14	0.23						
15	Mentha-2,4,8-Diene (*p*)	1098	1085	0.23			0.48		0.22			0.13	0.41	0.79	0.15	0.1
16	Terpinoline	1097	1086			0.36						0.87				
17	*p*-1,3,8-Menthatriene	1097	1108						0.48	0.3						
18	*Cis* Limonene Oxide	1129	1132	0.18								0.13				
19	Borneol	1170	1165	1.8	1.01	1	1.82	1.11	1.81	2	1.54	1.89	1.6	2.19	1.15	2.3
20	*m*-Cymen-8-ol	1176	1176	0.81	0.41						0.22	0.13	0.14			0.2

21	*p*-Cymen-8-ol	1182	1179	0.15												
22	α-Terpineol	1190	1186	0.4	0.29						0.19	0.17		0.86		
23	Verbenone	1208	1204	0.92	0.53	0.48			0.06	0.08		1.08	0.98	0.9	0.07	0.44
24	4-Methylene-Isophorone	1215	1216	0.21												
25	*Endo*-Fenchyl Acetate	1213	1218									0.13			0.03	0.1
26	Car-3-en-2-one	1242	1244	0.3												
27	Sabinyl Acetate (*trans*)	1288	1289								0.26					
28	*p*-Vinyl Guaiacol	1302	1309	0.3							0.64					
29	Myrtenyl Acetate	1323	1324								0.32					
30	α-Terpinyl Acetate	1350	1346	1.01	1	1					0.98	1.08	1.01	0.69	0.89	0.12
31	α-Longipinene	1357	1350	0.13												
32	β-Elemene	1383	1389					0.13								
33	Cyperene	1394	1398				1.1									
34	*n*-Tetradecane	1396	1400													
35	α-Gurjunene	1402	1409	1.19	1	1	1.01	1	1.31	1.16	1.03	1.12	1.04	1	1.03	1.3
36	β-Cedrene	1403	1419					0.16	0.22	0.33		0.78				
37	α-Guaiene	1431	1437							0.07		0.14		0.78	0.32	0.21
38	α-Humulene	1451	1452						0.14	0.21		0.98				
39	Ethyl Cinnamate	1467	1465	20.9	28	27	23.03	27.2	28.2	28.56	28.74	29.14	26.16	24.14	27.67	27
40	*Cis*-Muurola-4(14),5-Diene	1475	1465													
41	γ-Muurolene	1476	1478													
42	Germacrene D	1673	1484						0.09	0.2	0.19	0.15	0.31	0.91	0.76	0.17
43	δ-Selinene	1494	1492							0.37						
44	Pentadecane	1504	1500	12.5	10	12	11.98	24.93	10.6	18.01	10.33	9.09	12.47	8.78	7.14	11.78
45	Epizonarene	1508	1501								0.5					
46	γ-Cadinene	1513	1513	0.7	0.22	0.29	0.63	0.58	0.54	0.63		0.78	0.68	0.88	0.31	0.16
47	Cubebol	1523	1514								0.37					
48	β-Curcumene	1517	1514	0.4				0.13	0.47	0.71		0.31	0.16		0.31	0.21
49	δ-Cadinene	1529	1522		0.38	0.27	0.53	0.36		0.11						
50	α-Cadinene	1526	1537		0.4		0.36		0.09							
51	α-Copaene-11-ol	1540	1539								0.18					
52	Selina3,7(11)-Diene	1555	1545	0.7		0.29		0.06	0.17	0.2		0.1	0.2	0.87	0.25	0.14
53	Guaiol	1606	1594								0.19					
54	Carotol	1606	1594													
55	*Epi*-α-muurolol	1646	1640													
56	α-Muurolol	1651	1644	1.16	1	0.81	0.59		0.3	0.06	0.41	0.13	0.09	0.9	0.6	0.1

57	Pogostol	1662	1651													
58	Citronellyl Tiglate	1669	1666					0.1								
59	8,9 Epoxide Cadalene	1677	1674	1.06	0.37				0.81	1.08		1.11		1.21		0.86
60	Germacrone	1673	1693	1		0.45		1								
61	*n*-Heptadecane	1694	1700	0.44	0.26			0.71			0.36					
62	Zerumbone	1720	1732													
63	Ethyl-*p*-Methoxy Cinnamate	1769	1760	21.06	27	26	19.58	17.66	24.56	10.7	23.54	17.87	20.14	18.14	15.99	17.56
64	*n*-Pentadecanol	1773	1773	0.22												
65	Pimaradiene	1950	1948	0.3												
	Total Identified (%)			88.94	92.18	88.86	87.8	91.11	93.01	91.06	87.58	90.09	84.1	80.98	76.16	81.62
	Monoterpene Hydrocarbons			16.47	15.31	14.27	21.87	14.49	18.73	21.08	13.97	17.8	17.61	14.74	14.52	12.29
	Oxygenated Monoterpenes			10.52	8.24	6.48	7.17	2.73	6.78	7.61	7.71	10.75	5.82	7.85	7.29	9.7
	Sesquiterpene Hydrocarbons			3.12	2	1.85	3.63	2.42	3.03	3.99	1.72	4.36	2.39	4.44	2.98	2.19
	Oxygenated Sesquiterpens			3.44	1.37	1.26	0.59	1.1	1.11	1.14	1.15	1.24	0.09	2.11	0.6	0.96
	Alkane Hydrocarbones			12.94	10.26	12	11.98	25.64	10.6	18.01	10.69	9.09	12.47	8.78	7.14	11.78
	Polypropanoids			41.96	55	53	42.61	44.86	52.76	39.26	52.28	47.01	46.3	42.28	43.66	44.56

(c)

Sl no.	Compound	RI[a]	RI[b]	Assam							
				K.g10	K.g19	K.g20	K.g31	K.g32	K.g33	K.g34	K.g35
1	Tricyclene	922	921						0.15	0.11	
2	α-Thujene	923	924								
3	α-Pinene	932	932	1.72	1	1	1.2	1.45	2.1	1.98	1.1
4	Camphene	949	946	2.31	4.32	2.99	3.7	2	2.5	4.65	3.1
5	Sabinene	970	969								
6	β-Pinene	977	974	1.01	1.23	1.14	1.01	1.15	1.31	1.16	1.2
7	Myrecene	987	988	0.4	0.5	0.14	0.25	0.89	0.32	0.94	0.8
8	Dehydroxy *Trans* Linalool Oxide	993	991		0.87		0.1	0.2			
9	δ-Carene	1011	1008	11.76	10.5	9.87	11.2	7.1	8.13	9.45	10.13
10	*p*-Cymene	1021	1020			0.43		0.21	0.16	0.31	1.9
11	*o*-Cymene	1023	1022	1.07	1.1	1.01	1.09	1.21	1.16	1.31	1.9
12	Limonene	1028	1024	1.08	1.19	1	1.03	1.16	1.31	1.22	1.7
13	1,8-Cineol	1032	1026	3.51	5.21	3.21	3.76	4.64	4.12	3.99	3.5
14	γ-Terpinene	1054	1054								
15	Mentha-2,4,8-Diene (*p*)	1098	1085		0.23					0.12	0.31
16	Terpinoline	1097	1086								
17	*p*-1,3,8-Menthatriene	1097	1108								

18	*Cis* Limonene Oxide	1129	1132								
19	Borneol	1170	1165	1.6	1.6	1.46	1.24	1.31	1.65	1.76	1.58
20	*m*-Cymen-8-ol	1176	1176								
21	*p*-Cymen-8-ol	1182	1179								
22	α-Terpineol	1190	1186			0.11	0.12	0.26	0.44	0.16	0.19
23	Verbenone	1208	1204								
24	4-Methylene-Isophorone	1215	1216								
25	*Endo*-Fenchyl Acetate	1213	1218								
26	Car-3-en-2-one	1242	1244								
27	Sabinyl Acetate (*trans*)	1288	1289								
28	*p*-Vinyl Guaiacol	1302	1309								
29	Myrtenyl Acetate	1323	1324								
30	α-Terpinyl Acetate	1350	1346		0.4	0.23	1	0.89	0.67	0.22	
31	α-Longipinene	1357	1350								
32	β-Elemene	1383	1389			0					
33	Cyperene	1394	1398								
34	*n*-Tetradecane	1396	1400								
35	α-Gurjunene	1402	1409	1.56	1.01	1.23	1	1	1.14	1.09	1.2
36	β-Cedrene	1403	1419	0.48		0.54	0.87	0.32		0.13	
37	α-Guaiene	1431	1437								
38	α-Humulene	1451	1452	0.24	0.1			0.4			
39	Ethyl Cinnamate	1467	1465	24.9	25.8	23.89	23.16	24.17	21.54	25.02	26
40	*Cis*-Muurola-4(14),5-Diene	1475	1465			0					
41	γ-Muurolene	1476	1478								
42	Germacrene D	1673	1484		0.16	0.59		0.11	0.47	0.21	0.13
43	δ-Selinene	1494	1492								
44	Pentadecane	1504	1500	14.52	10.12	11.54	13.13	12.46	11.24	8.14	12.1
45	Epizonarene	1508	1501			0					
46	γ-Cadinene	1513	1513	0.66	0	0		0.56	0.66	0.21	0.1
47	Cubebol	1523	1514								
48	β-Curcumene	1517	1514	0.5	0.14	0.65	0.15	0.31	0.42	0.13	0.4
49	δ-Cadinene	1529	1522	0.26	0.13	0.89	0.3	0.3	0.65	0.6	0.14
50	α-Cadinene	1526	1537								
51	α-Copaene-11-ol	1540	1539		0.21						
52	Selina3,7(11)-Diene	1555	1545	0.31			0.21	0.31	0.43	0.15	0.1
53	Guaiol	1606	1594								

54	Carotol	1606	1594								
55	*Epi*-α-Muurolol	1646	1640								
56	α-Muurolol	1651	1644	0.39	0.16	0.33	0.31	0.22	0.33	0.19	0.11
57	Pogostol	1662	1651								
58	Citronellyl Tiglate	1669	1666								
59	8,9 Epoxide Cadalene	1677	1674	0.86	0.17		0.71	0.31	0.56	0.11	0.89
60	Germacrone	1673	1693								
61	*n*-Heptadecane	1694	1700		0.12				0.61		
62	Zerumbone	1720	1732								
63	Ethyl-*p*-Methoxy Cinnamate	1769	1760	22.21	18.14	20.14	19.18	20.14	19.43	17.16	19.1
64	*n*-Pentadecanol	1773	1773								
65	Pimaradiene	1950	1948								
	Total Identified (%)			91.35	84.41	82.39	84.72	83.08	81.5	80.52	87.68
	Monoterpene Hydrocarbons			19.35	20.07	17.58	19.48	15.32	17.1	21.14	22.14
	Oxygenated Monoterpenes			5.11	8.08	5.01	6.22	7.3	6.88	6.13	5.27
	Sesquiterpene Hydrocarbons			4.01	1.54	3.9	2.53	3.31	3.77	2.52	2.07
	Oxygenated Sesquiterpens			1.25	0.54	0.33	1.02	0.53	0.89	0.3	1
	Alkane Hydrocarbones			14.52	10.24	11.54	13.13	12.46	11.85	8.14	12.1
	Polypropanoids			47.11	43.94	44.03	42.34	44.31	40.97	42.18	45.1

[a] RI, Retention indices on Elite-5 column, experimentally determined using homologous series of C8–C20 *n*-alkanes. [b] RI; Retention index taken from literature [30].

Table S2. Chemical Structure of all identified constituents from *K. galanga* rhizome essential oil.

Sl.No.	Compounds Name	Structure
1	Tricyclene	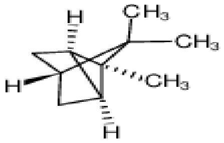
2	α-Thujene	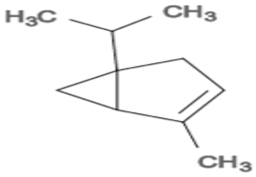
3	α-Pinene	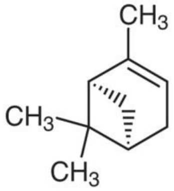
4	Camphene	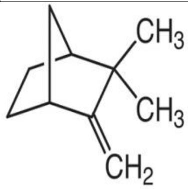
5	Sabinene	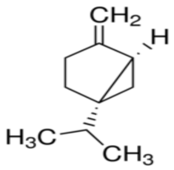
6	β-Pinene	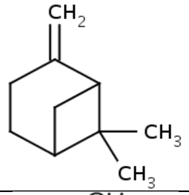
7	Myrecene	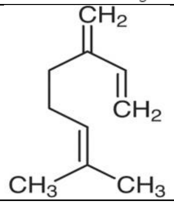
8	Dehydroxy *Trans* Linalool Oxide	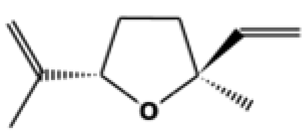
9	δ-Carene	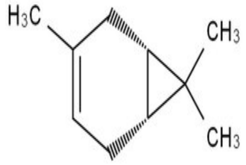

10	*p*-Cymene	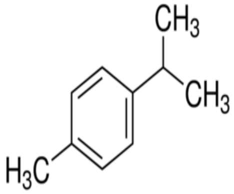
11	*o*-Cymene	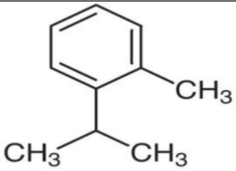
12	Limonene	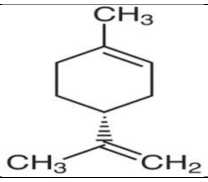
13	1,8-Cineol	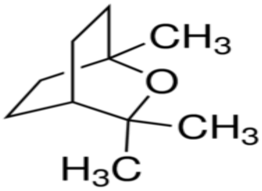
14	γ-Terpinene	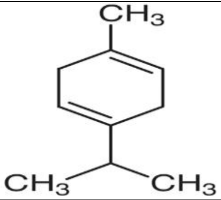
15	Mentha-2,4,8-Diene (*p*)	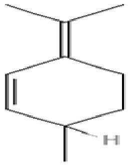
16	Terpinoline	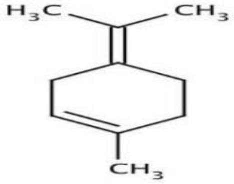
17	*p*-1,3,8-Menthatriene	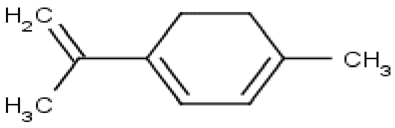
18	*Cis* Limonene Oxide	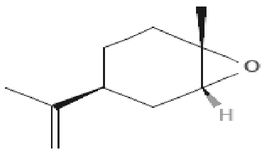

19	Borneol	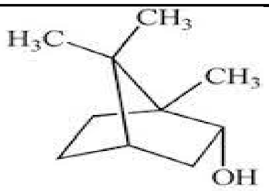
20	*m*-Cymen-8-ol	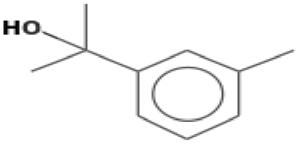
21	*p*-Cymen-8-ol	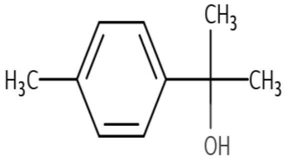
22	α-Terpineol	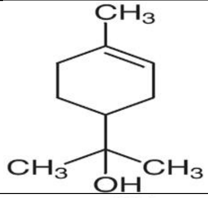
23	Verbenone	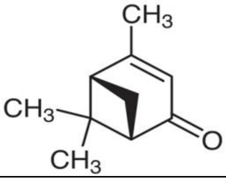
24	4-Methylene-Isophorone	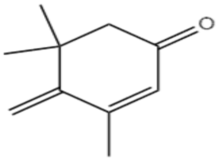
25	*Endo*-Fenchyl Acetate	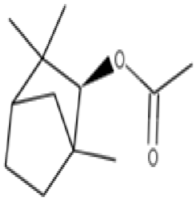
26	Car-3-en-2-one	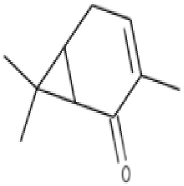
27	Sabinyl Acetate (*trans*)	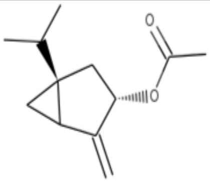

28	*p*-Vinyl Guaiacol	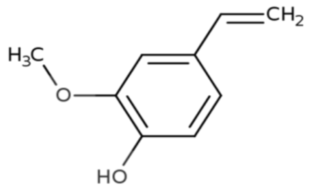
29	Myrtenyl Acetate	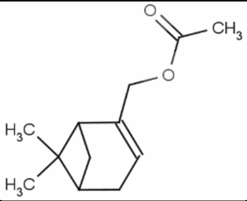
30	α-Terpinyl Acetate	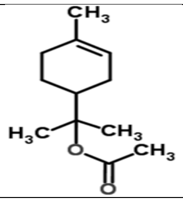
31	α-Longipinene	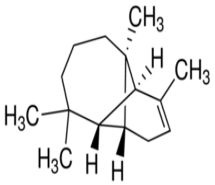
32	β-Elemene	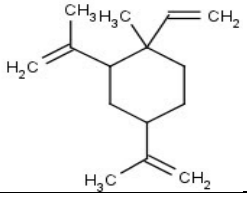
33	Cyperene	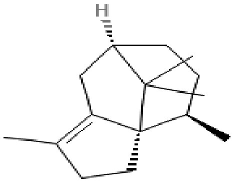
34	*n*-Tetradecane	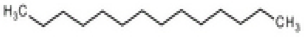
35	α-Gurjunene	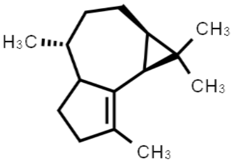
36	β-Cedrene	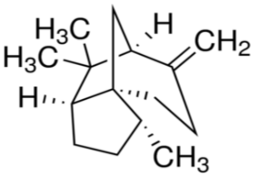

37	α-Guaiene	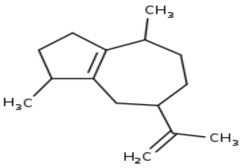
38	α-Humulene	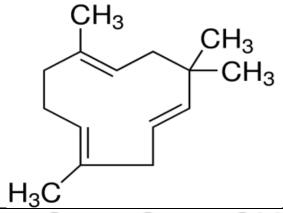
39	Ethyl Cinnamate	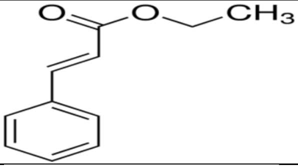
40	*Cis*-Muurola-4(14),5-diene	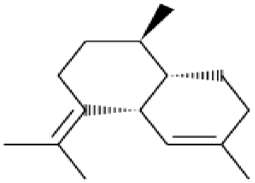
41	γ-Muurolene	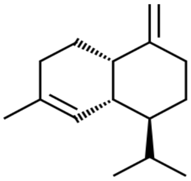
42	Germacrene D	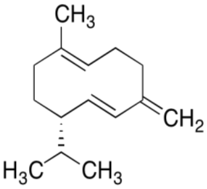
43	δ-Selinene	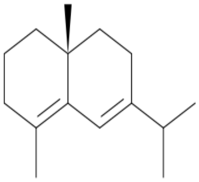
44	Pentadecane	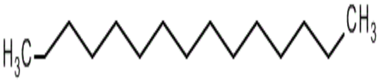
45	Epizonarene	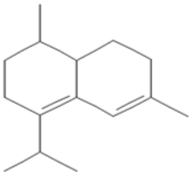

46	γ-Cadinene	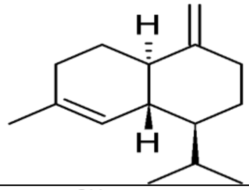
47	Cubebol	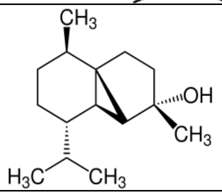
48	β-Curcumene	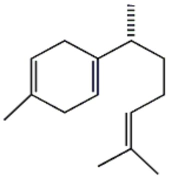
49	δ-Cadinene	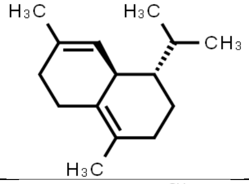
50	α-Cadinene	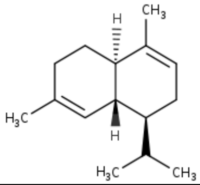
51	α-Copaene-11-ol	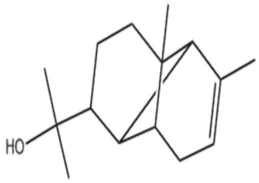
52	Selina3,7(11)-Diene	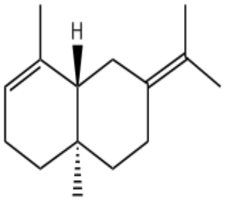
53	Guaiol	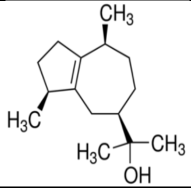
54	Carotol	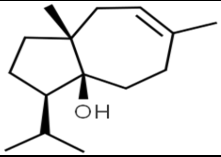

55	*Epi*-α-Muurolol	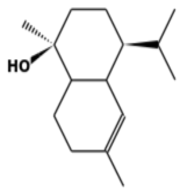
56	α-Muurolol	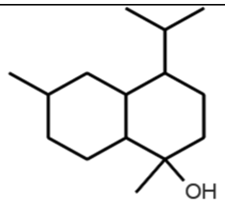
57	Pogostol	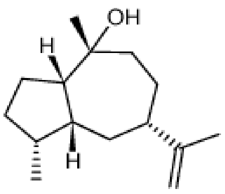
58	Citronellyl Tiglate	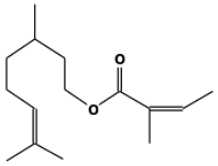
59	8,9 Epoxide Cadalene	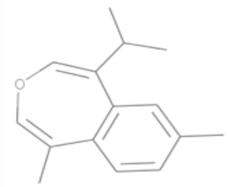
60	Germacrone	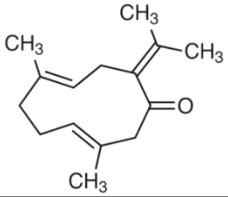
61	*n*-Heptadecane	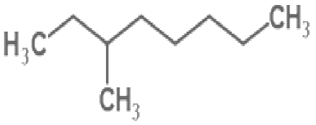
62	Zerumbone	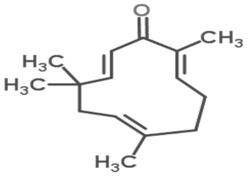
63	Ethyl-*p*-Methoxy Cinnamate	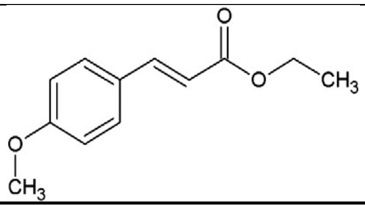

64	*n*-Pentadecanol	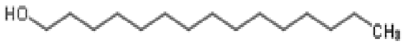
65	Pimaradiene	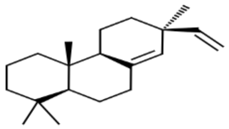

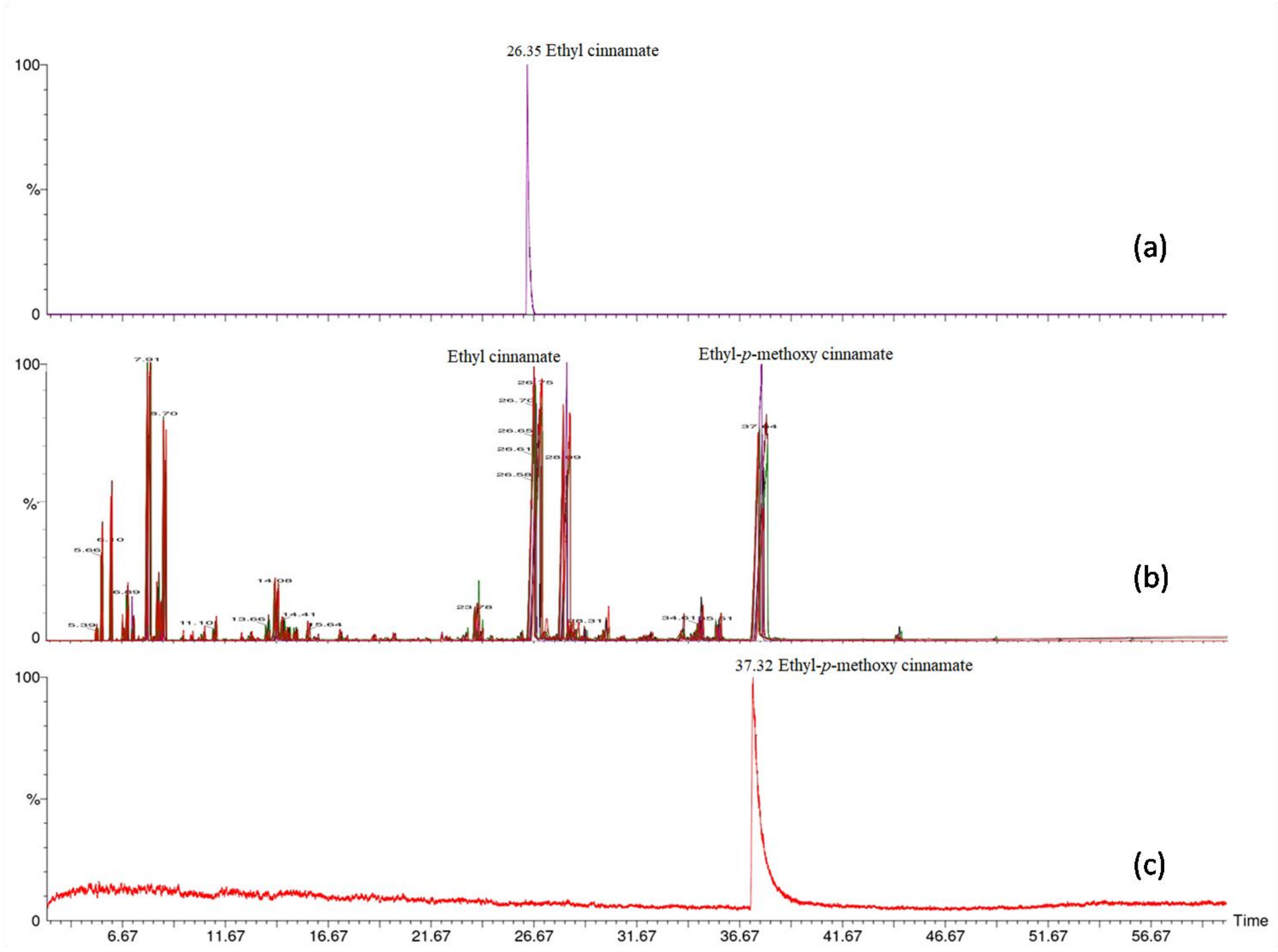

Figure S1. Authentic identification of EC and EPMC constituents in analytes by co-injecting with known standards through GC-MS. (**a**) Chromatogram of EC standard (**b**) Consensus GC-MS fingerprint of *K. galanga* accessions collected from three different provinces showing similar pattern. (**c**) Chromatogram of EPMC standard.
